# 1122. Case Study. RN Quality Review Integration to Burn Surgery Service Line Quality Improvement Process

**DOI:** 10.1093/jbcr/irag033.474

**Published:** 2026-04-06

**Authors:** Nikole Mercedes Williams, Rachael A Underwood

**Affiliations:** University of Utah Health Burn Center, Salt Lake City, UT; University of Utah Health Burn Center, Salt Lake City, UT

## Abstract

**Patient Presentation (age range, injury details, relevant history):**

Based on multi-patient practice gap identification.

In 2025 a poster presentation was accepted for implementation of an RL-lead quality review committee. We have since received multiple requests from our hospital and burn centers for assistance in implementation of a like-committee. Where this process is easily replicated and has many successful examples, the hope is to have an opportunity for discussion with other burn centers interested in implementation or have recently initiated this workflow and want to discuss the process. Focus on the quality improvement loop closure and process efficacy tracking that this process provides a clear and direct space for would be the primary topic of discussion.

**Clinical Challenges:**

Designating appropriate stakeholders in each department to review sensitive information within the reporting system. Having transparency with quality improvement while being sensitive to practitioner concerns through and interdisciplinary team requires intentional selection of key stakeholders.

**Management Approach:**

The committee is comprised of RN lead, Shared governance committee chair, unit nurse management, APC representative, and IDT members. Monthly meeting with selected incident reports and debriefing tools reviewed. Each report is documented and scored using the trauma PIPS scoring scale and referred to appropriate project committee or M&M review. Projects are tracked for efficacy by management.

**Outcomes:**

Management is how able to track incident reports into themes that line up with burn center process improvement practices. This allows us to track and pull data on an ongoing basis and identify gaps sooner, leading to better patient outcomes. The practice of reviewing debriefing tools allows burn management and senior staff to follow up with staff members that may need support or positive shoutouts after debriefing. Since our poster presentation in 2025, we have escalated 4 RN Quality review cases to Burn Surgery M&M and received 5 case reviews from M&M with which we returned with recommendations and plan for improvement. We have had 3 burn centers reach out for guidance in implementing an RN-lead quality review committee at their burn center. The efficacy of this process has been proven and the desire to replicate it has been far reaching.

**Lessons Learned:**

Implementing this process within an existing workflow has created trust within the team to assess opportunities for improvement and come to the table with recommendations or plans. This has made M&M more efficient by decreasing the agenda items marked for loop closure and given space for providing loop closure to staff. Having such thorough review of processes has lead to increase investment in our reporting structure per survey results. Hope for the future is having a clear workflow we can teach and mentor to will build a more robust team with increased number of future leaders.

**Applicability to Practice:**

The goal of this workflow is to apply research to practice in a proactive approach by identifying trends early and updating established workflows to align with best practice. An example of this is applying the practice of a slower PACU recovery of pediatric patients. This committee responded to the need for nursing comfortability with pediatric airway management in a PACU setting by delegating this education need to our Skin Bud team who created a pediatric airway station. The following month we had an increase in pediatric PACU patients with lower Riker scores, and no reports related to concerns with comfortability in maintaining open airways during recovery. This process allowed us to implement best PACU practice at the bedside in a safe way. Buy-in at the surgeon level has added to the efficacy of this process as cases are reviewed in IDT M&M and sent to RN quality when it is identified that nursing needs to review this case and come back to IDT M&M with an improvement plan.

